# Exploring the shared molecular mechanism of microvascular and macrovascular complications in diabetes: Seeking the hub of circulatory system injury

**DOI:** 10.3389/fendo.2023.1032015

**Published:** 2023-01-23

**Authors:** Cao Yuchen, Zhao Hejia, Meng Fanke, Deng Qixin, Cai Liyang, Guo Xi, Chen Yanxia, Yang Xiongyi, Xie Zhuohang, Yi Guoguo, Fu Min

**Affiliations:** ^1^ Department of Ophthalmology, Zhujiang Hospital, Southern Medical University, Guangzhou, China; ^2^ The Second Clinical School, Southern Medical University, Guangzhou, China; ^3^ Plastic Surgery Hospital, Peking Union Medical College, Chinese Academy of Medical Sciences, Beijing, China; ^4^ School of Public Health, Southern Medical University, Guangzhou, China; ^5^ Department of Emergency, Zhujiang Hospital, Southern Medical University, Guangzhou, China; ^6^ Department of nephrology, The First Affiliated Hospital, Sun Yat-sen University, Guangzhou, China; ^7^ School of Rehabilitation Sciences, Southern Medical University, Guangzhou, Guangdong, China; ^8^ School of Psychological and Cognitive Sciences and Beijing Key Laboratory of Behavior and Mental Health, Peking University, Beijing, China; ^9^ Department of Ophthalmology, The Sixth Affiliated Hospital, Sun Yat-sen University, Guangzhou, China

**Keywords:** atherosclerosis, diabetic retinopathy, diabetic nephropathy, complications of circulatory system, inflammation-oxidative stress, immune regulation, bioinformatics cross analysis

## Abstract

**Background:**

Microvascular complications, such as diabetic retinopathy (DR) and diabetic nephropathy (DN), and macrovascular complications, referring to atherosclerosis (AS), are the main complications of diabetes. Blindness or fatal microvascular diseases are considered to be identified earlier than fatal macrovascular complications. Exploring the intrinsic relationship between microvascular and macrovascular complications and the hub of pathogenesis is of vital importance for prolonging the life span of patients with diabetes and improving the quality of life.

**Materials and methods:**

The expression profiles of GSE28829, GSE30529, GSE146615 and GSE134998 were downloaded from the Gene Expression Omnibus database, which contained 29 atherosclerotic plaque samples, including 16 AS samples and 13 normal controls; 22 renal glomeruli and tubules samples from diabetes nephropathy including 12 DN samples and 10 normal controls; 73 lymphoblastoid cell line samples, including 52 DR samples and 21 normal controls. The microarray datasets were consolidated and DEGs were acquired and further analyzed by bioinformatics techniques including GSEA analysis, GO-KEGG functional clustering by R (version 4.0.5), PPI analysis by Cytoscape (version 3.8.2) and String database, miRNA analysis by Diana database, and hub genes analysis by Metascape database. The drug sensitivity of characteristic DEGs was analyzed.

**Result:**

A total of 3709, 4185 and 8086 DEGs were recognized in AS, DN, DR, respectively, with 1820, 1666, 888 upregulated and 1889, 2519, 7198 downregulated. GO and KEGG pathway analyses of DEGs and GSEA analysis of common differential genes demonstrated that these significant sites focused primarily on inflammation-oxidative stress and immune regulation pathways. PPI networks show the connection and regulation on top-250 significant sites of AS, DN, DR. MiRNA analysis explored the non-coding RNA upstream regulation network and significant pathway in AS, DN, DR. The joint analysis of multiple diseases shows the common influenced pathways of AS, DN, DR and explored the interaction between top-1000 DEGs at the same time.

**Conclusion:**

In the microvascular and macrovascular complications of diabetes, immune-mediated inflammatory response, chronic inflammation caused by endothelial cell activation and oxidative stress are the three links linking atherosclerosis, diabetes retinopathy and diabetes nephropathy together. Our study has clarified the intrinsic relationship and common tissue damage mechanism of microcirculation and circulatory system complications in diabetes, and explored the mechanism center of these two vascular complications. It has far-reaching clinical and social value for reducing the incidence of fatal events and early controlling the progress of disabling and fatal circulatory complications in diabetes.

## 1 Introduction

Diabetes, as the ninth leading cause of death, has also been identified as one of the four major non-infectious diseases by the World Health Organization (WHO), which threatens human life and deserves close attention (1). Globally, the number of patients with diabetes has quadrupled in the past 30 years. About one in ten adults worldwide suffer from diabetes (2), of which 90% are type 2 diabetes (T2DM) (3, 4). Between 2005 and 2019, the number of deaths due to diabetes increased by 48%, from 2 million to 3 million (5), with the main cause of cardiovascular complications (6).

Complications of diabetes are very common. Traditionally, they can be divided into macrovascular complications and microvascular complications. Macrovascular diseases mainly refer to cardiovascular and cerebrovascular diseases, including atherosclerosis (AS), coronary heart disease, etc. Whereas, microvascular diseases mainly refer to diabetic retinopathy (DR), diabetic nephropathy (DN) and diabetic foot. Studies have indicated that the risk of macrovascular disease in diabetes patients is at least 2-4 times higher than that in non-diabetes patients (7). Therefore, more than 30% of hospitalized patients with acute myocardial infarction have diabetes, and more than 35% have abnormal glucose tolerance (8). Simultaneously, the prevalence of microvascular disease in patients with diabetes is about 10-20 times higher than that in healthy people (7). While about 10% of diabetes patients die of renal failure, DN is also a microvascular complication that deserves special attention (9). Whether in Europe and the United States or Asian countries, diabetes related chronic kidney disease has become the main cause of end-stage renal disease (ESRD) (10, 11). DR is another microvascular complication that seriously affects the patients’ quality of life. In the United States, the prevalence of DR is about 28.5% (12), while in Asian countries, the prevalence is between 16% and 35% (13). The irreversibility of the course of DR will finally deprive patients of vision at the end stage. In conclusion, microcirculation complications and cardiovascular and cerebrovascular diseases will seriously reduce the quality of life, shorten the lifespan and increase social pressure.

With the gradual deepening of the research on diabetes, many evidence supports that hyperglycemia causes tissue damage mainly through five mechanisms: (1) increased intracellular formation of advanced glycation end-products (AGEs); (2) increased expression of the receptor for advanced glycation end products and its activating ligands; (3) increased flux of glucose and other sugars through the polyol pathway; (4) activation of protein kinase C (PKC) isoforms; and (5) overactivity of the hexosamine pathway. Plenty lines of evidence signified that these five mechanisms seem to be regulated upstream by a large amount of ROS produced by mitochondria. Some studies have pointed out that in the microvascular system, this is the result of intracellular hyperglycemia. In contrast, in macro-vessels and heart complications, this seems to be the result of increased fatty acid oxidation, partly due to pathway specific insulin resistance (14).

In order to find the intrinsic relationship between diabetes related microvascular and macrovascular complications, we download data from GEO database, including microvascular complications (DR, DN) and macrovascular complications (AS), compare and crosslink the three diseases, and construct diseases cross-linking network. Furthermore, we determine the upstream mi-RNA regulatory network, and then try to identify common differentially expressed genes and pathways as diagnostic biomarkers of patients. Providing easier access to the diagnosis and treatment of circulatory complications of diabetes, this study may facilitate doctors finding the underlying high-risk factors of fatal cardiovascular and cerebrovascular complications when non-invasive routine screening projects such as fundus photography and routine urine testing are abnormal, and finally better manage diabetes, reduce the disability and mortality rate as well as relieve social burden.

## 2 Method and materials

### 2.1 Microarray data

The gene expression data was downloaded from the free public database Gene Expression Omnibus (GEO) database (http://www.ncbi.nlm.nih.gov/geo). “Diabetic Retinopathy”, “Diabetes nephropathy” and “Atherosclerosis” were set as the keywords to search in the GEO database

### 2.2 Gene expression data sources

The gene expression data was downloaded from the free public database Gene Expression Omnibus (GEO) database (http://www.ncbi.nlm.nih.gov/geo). Raw gene expression profiling of DR, DN, AS patients was accessed from GSE146615 (15), GSE30529 (16), GSE28829 (17) and GSE134998 (18) datasets. In the GSE146615 dataset, there are 41 samples of lymphoblastoid cell lines (LCLs) from diabetic individuals without retinopathy and 103 samples of LCLs from individuals with diabetic retinopathy, detected by the Illumina HumanHT-12 V4.0 expression beadchip. Among them, we selected 21 samples from diabetic individuals without retinopathy and 52 samples from individuals with diabetic retinopathy. The GSE30529 dataset includes 10 microdissected human kidney samples of diabetic Human Kidney and 12 microdissected human kidney samples of non-diabetic Human Kidney, detected by [HG-U133A_2] Affymetrix Human Genome U133A 2.0 Array. The GSE28829 dataset contains 13 early carotid atherosclerotic plaque specimens (pathological intimal thickening and intimal xanthoma) and 16 advanced carotid atherosclerotic plaque specimens (thin or thick fibrous cap atheroma), detected by the Affymetrix Human Genome U133 Plus 2.0 Array. The GSE134998 dataset comprises 15 newly diagnosed untreated diabetic cases and 15 healthy controls, detected by miRNA array screening and Taqman real-time PCR validation, Exiqon miRCURY LNA microRNA array, 7th generation [miRBase v18, condensed Probe_ID version]. The mRNA expression profile microarrays were used for Gene difference analysis for DR, DN, AS. MiRNA sequencing data set for screening upstream regulators of disease pathways. The probe ID for each gene was transformed into a gene symbol. If a gene symbol corresponded to several probe IDs, the average expression value of the probe IDs was calculated as the representative expression value of the gene.

### 2.3 Identification of differentially expressed genes

Data quality checking and normalization with log transformation were first performed to eliminate any batches. The ‘limma’ package (19) in R software (v4.1.1, Vienna, VIE, Austria) was employed to screen DEGs between the DR, DN, AS and control group. An adjusted P-value < 0.05 was set as the threshold criterion for statistical significance. The volcano map of DEGs was plotted in R software. The Heatmap package in R software was utilized to visualize the top 50 DEGs.

### 2.4 Functional enrichment analysis

ClusterProfiler package was utilized for functionally analyzing the biological functions, which comprises Gene Ontology (GO) and Kyoto Encyclopedia of Genes and Genomes (KEGG). The P-value was adjusted using the Benjamini–Hochberg approach or FDR for multiple testing corrections. The threshold was set at FDR<0.05. GO categories comprised biological processes (BP), molecular functions (MF), and cellular components (CC) (**SFigure 1**).

### 2.5 Protein-protein interaction network analysis and sub-network construction

To explore the interaction of DR, DN, and AS, Gene differential expression lists of DR, DN, AS were first imported into STRING database (https://string-db.org) (20), and generated the protein-protein interaction (PPI) network. When constructing the PPI network, protein-protein interaction pairs with medium confidence of interaction score > 0.4 were exported and then visualized by using Cytoscape (version 3.4.0) (21). To analyze the topological structure and relationship in PPI network, CentiScaPe was further applied for calculating centrality parameters for each node and finding the most important nodes in a network. At last, Molecular COmplex Detection (MCODE) (http://apps.cytoscape.org/apps/mcode) was applied to find densely connected regions and sub-networks within the main network.

### 2.6 Upstream regulatory miRNA analysis

MiRNA analysis is mainly based on the difference analysis of diabetes human plasma miRNA sequencing data set GSE134998, as well as the downstream site analysis of the top 100 miRNAs and the GO-KEGG functional cluster analysis of the top 50 miRNAs. Among them, mirExTra tools (https://dianalab.e-ce.uth.gr/html/mirextra/index.php?r=mirExTra) of DIANA database were used to do the analysis of miRNA downstream sites, and the functional clustering analysis mainly adopts mirPath v.3 (https://dianalab.e-ce.uth.gr/html/mirpathv3/index.php?r=mirpath) of Diana database.

### 2.7 Joint analysis and GSEA of multiple diseases

The significantly different genes of DR, DN and AS were jointly analyzed, and the common heterotopic sites were selected. The results were visualized in Rstdio v4.0.5 by R-package Venndiagram.

Gene Set Enrichment Analysis (GSEA) (22) was applied to detect whether the enrichment of the KEGG pathway is statistically significant in AMI patients and controls. Transcriptome data were imported into the GSEA desk application strictly according to the website instruction. Both p < 0.05 and FDR < 0.25 were considered as the criteria for the significant gene sets.

### 2.8 Aggregation analysis of multiple diseases

Select the top 1000 significant difference outliers of the three diseases for analysis. Gene ontology (GO) and enrichment analyses of time-dependent DEGs were performed using Metascape (http://metascape.org/gp/index.html accessed on 6 February 2021) (23). The Metascape analysis workflow followed these criteria. First, the multiple gene lists identified from DEG analysis were used as input genes. Second, for GO annotation, three main categories of gene functions (biological process (BP), molecular function (MF), and cellular component (CC)) were extracted. Third, functional enrichment analysis was performed with default parameters (min overlap of 3, enrichment factor of 1.5, and P-value of 0.01) for filtering (21). The Reactome pathway database, which interprets biological pathways, was also used to identify the functional role of genes that show differences in expression depending on injury periods. The protein–protein interaction (PPI) network was constructed and visualized using STRING software. At last, Molecular COmplex Detection (MCODE) (24) (http://apps.cytoscape.org/apps/mcode) was applied to find densely connected regions and sub-networks within the main network.

### 2.9 Determination of diabetes related genes

The central core sites in the PPI network of AS, DN and DR are selected and performed by the Attie Lab Diabetes database (http://diabetes.wisc.edu). The Attie lab diabetes database is a searchable resource of gene expression data for displaying gene expression profiles of different experimental groups (lean and obese BTBR mice at 4 and 10 weeks of age) in any of the six tissues, including islet and adipose (25).

### 2.10 Exploration of drug sensitivity for characteristic DEGs

The NCI-60 cell line panel was developed as drug efficacy screen by the developmental therapeutics program (DTP) of the US National Cancer Institute (NCI). Many thousands of compounds have been applied to the NCI-60. CellMiner (https://discover.nci.nih.gov/cellminer/) was a web-based suite of genomic and pharmacologic tools to explore transcript and drug patterns in the NCI-60 cell line set. The associations between the expression of six characteristic genes of diabetes (TYROBP, LCP2, CCL2, CD44, RPL3, CDK4) and drug sensitivity were performed by the Corrplot R package with the Spearman method (p < 0.05) based on the corresponding data from CellMiner.

## 3 Result

### 3.1 Identifications of DEGs for AS, DN and DR

**Figure 1:**
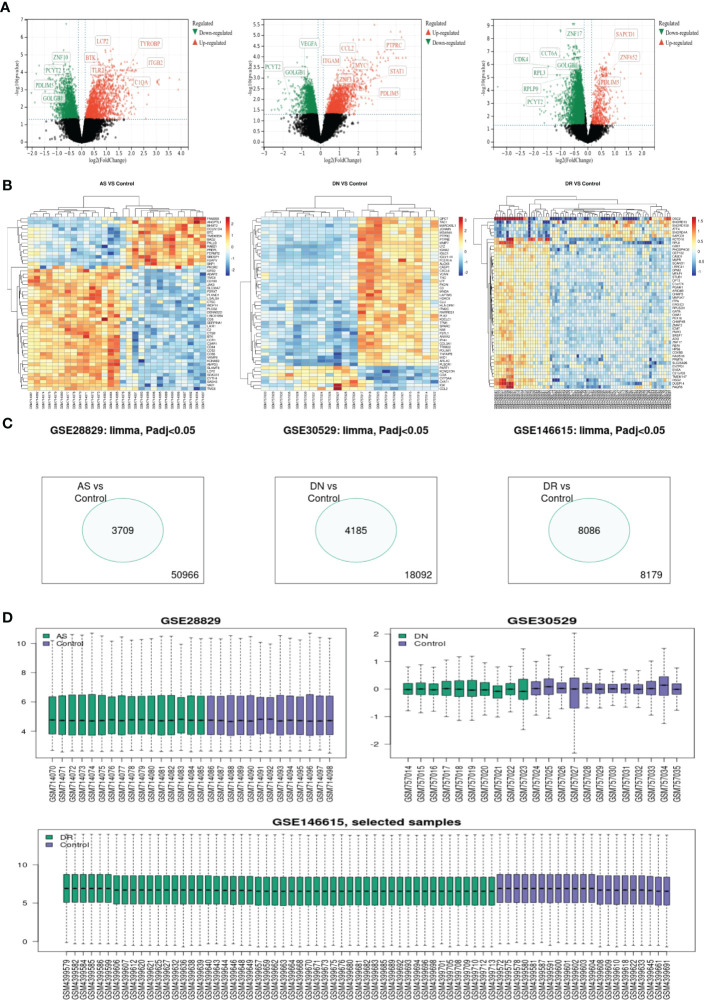
**(A)** Volcano map shows expression levels of DEGs from AS, DN and DR compared with the control group. **(B)** Heatmap of top 50 DEGs of AS, DN and DR, respectively. **(C)** Venn map shows significantly different genes among all genes involved in sequencing. **(D)** Box map of selected samples of AS, DN and DR.

A total of 3709, 4185 and 8086 differentially expressed genes were identified, with an adjusted p-value < 0.05 between AS, DN, and DR patients and each control sample. Of these 3 groups of DEGs, 1820 genes were upregulated and 1889 genes were downregulated in AS, 1666 genes were upregulated and 2519 genes were downregulated in DN, 888 genes were upregulated and 7198 genes were downregulated in DR. The volcano map for DEGs of AS, DN, DR is shown in **Figure 1A**. The heatmap for the top 50 DEGs is displayed in **Figure 1B**. The Venn map shows the DEGs with an adjusted p-value < 0.05 between AS, DN, and DR patients and each control sample in **Figure 1C**. The boxplot reveals the sample selection and grouping methods in **Figure 1D**.

### 3.2 PPI network construction, modular analysis, and hub gene analysis

**Figure 2:**
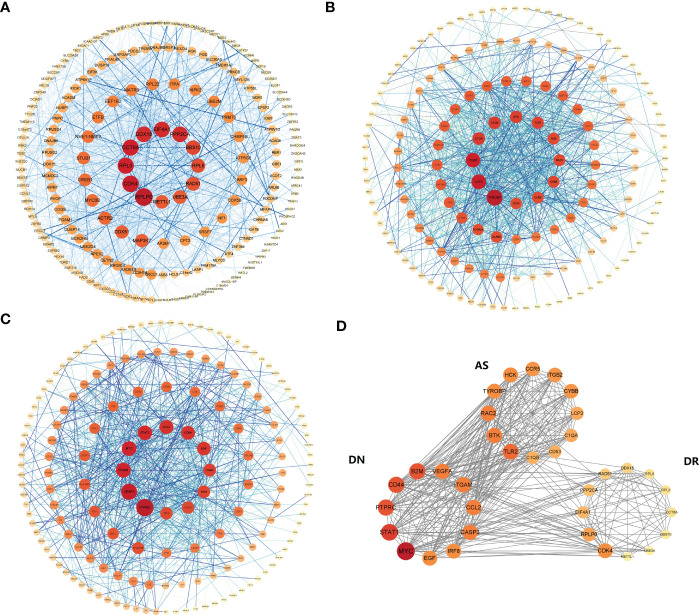
PPI network: **(A)** Top-250 sites filtered into the PPI network that contained 167 nodes and 1048 edges in AS. **(B)**Top-250 sites filtered into the PPI network that contained 166 nodes and 812 edges in DN. **(C)** Top-250 sites filtered into the PPI network that contained 204 nodes and 1232 edges in DR. **(D)** The connection of the three network (Constructed by String (version 11.5) and optimized by Cytoscape (version 3.8.2)).

To explore the interaction of top 250 genes sort by P-value from DEGs of AS, DN and DR, a protein–protein interaction network was constructed using the STRING database. Then, 0.4 was set as the threshold as the minimum required interaction score for constructing the STRING PPI network and hiding single and unconnected nodes to ensure the reliability of site interaction. As is shown in **Figure 2A**, the PPI network of top 250 genes in AS comprised 167 nodes and 1048 edges. Then in **Figure 2B**, the PPI network of top 250 genes in DN comprised 166 nodes and 812 edges. Additionally, in **Figure 2C**, the PPI network of top 250 genes in DR comprised 204 nodes and 1232 edges. Since then, we have connected the PPI network of three diseases in **Figure 2D**. The obvious relevance of PPI network indicates that there is a close molecular mechanism network among AS, DN and DR. Using the Analyze network tool in Cytoscape, we divided PPI networks into 4 sub circles, the core interaction network of the center can greatly reflect the interaction relationship between AS, DN, DR core proteins, which is conducive to exploring the molecular biological effects of disease and the intrinsic relationship of AS, DN and DR.

### 3.3 Upstream regulatory factors analysis

**Figure 3:**
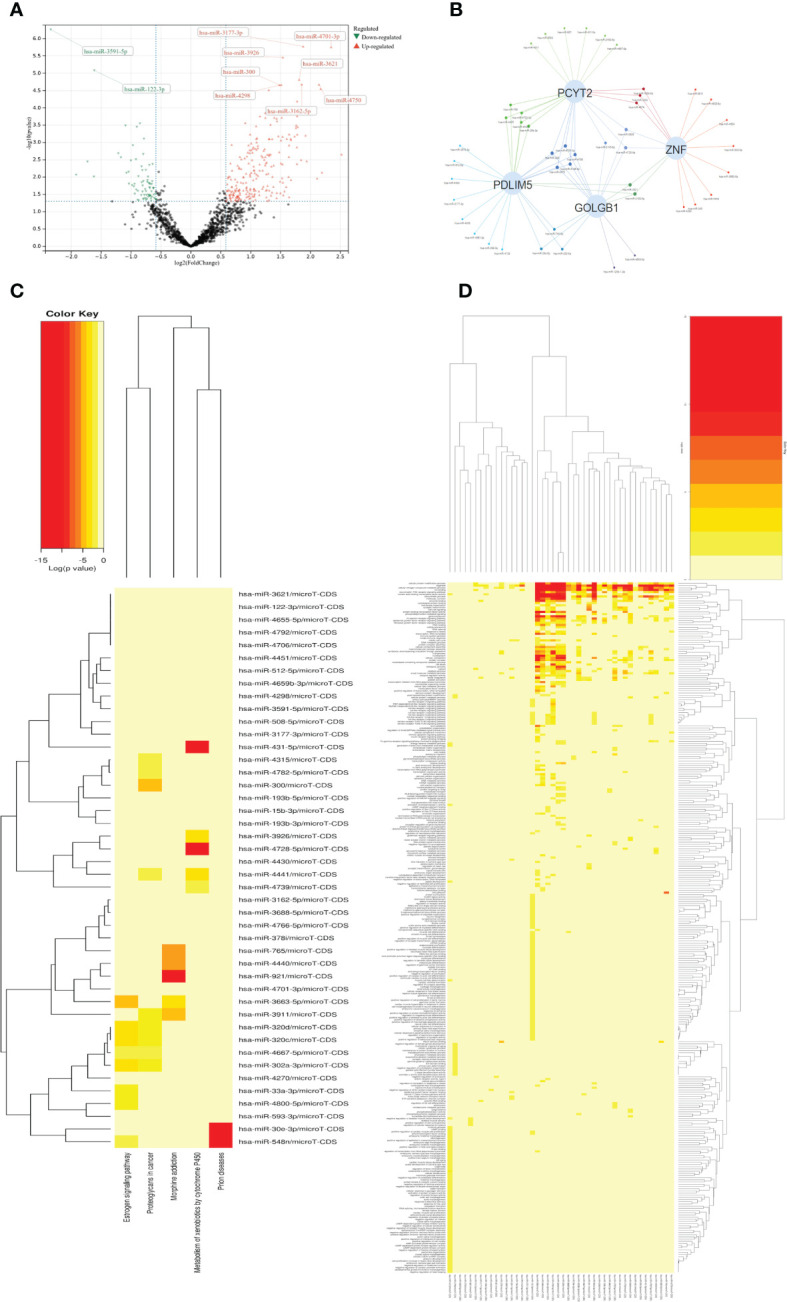
**(A)** Volcano map shows expression levels of differential MicroRNA from Diabetic dataset GSE134998. **(B)** Venn networks shows the interaction between four significant downstream sites (PCYT2、ZNF、PDLIM5、GOLGB1) from all three diseases and Top-100 MicroRNA. **(C)** Heatmap shows KEGG terms in the enrichment analysis of the Top-50 MicroRNA. **(D)** Heatmap shows GO terms in the enrichment analysis of the Top-50 MicroRNA.

By analyzing arrays of non-coding RNA profiling dataset GSE134998 of diabetes patients in GEO database, we constructed the upstream microRNA regulation community of diabetes patients, and obtained 333 significantly different microRNA by differential analysis using R-package limma of R-studio, including 88 down-regulation sites and 245 up-regulation sites. The results of the differential analysis were shown by volcano plot in **Figure 3A**. Top100 sites based on the significance of P-value were selected to analyze the common downstream regulatory sites, and the common significant differential sites of AS, DN, DR were selected to construct the regulatory network diagram of microRNA on the significant sites(PDLIM5, GOLGB1, ZNF, PCYT2). The results were shown by Network-Venn plot in **Figure 3B**. The four significant sites all have significant differences and have shown in the gene differentially expressed volcano plot (**Figure 1**). Then we selected the significantly expressed Top50 sites for GO-KEGG cluster analysis to clarify the functional mode of microRNA and the influence mode of downstream sites. The top50 sites were mainly enriched in Estrogen signaling pathway, Proteoglycans in cancer, Morphine addiction, Metabolism of xenobiotics by cytochrome P450, Prion diseases of KEGG pathway enrichment, innate immune response, immune system process, transcription, DNA-templated, response to stress, RNA binding, mRNA processing, RNA splicing, mitotic cell cycle, DNA metabolic process, protein complex assembly of GO terms. The results of GO terms and KEGG pathway enrichment were shown in **Figures 3C, D**

### 3.4 GSEA analysis for common differential genes

**Figure 4:**
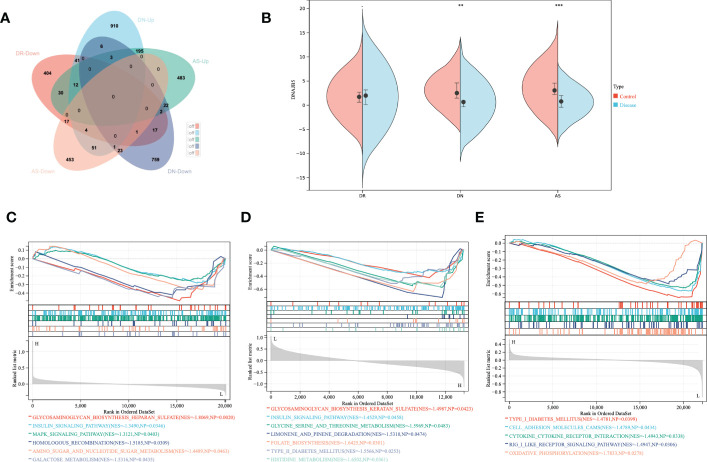
Single gene GSEA analysis of co-down-regulated genes. **(A)** The shared DEGs of AS, DN and DR. **(B)** Expression of DNAJB5 in AS, DN and DR. **(C)** The DNAJB5 single gene GSEA results of DR. **(D)** The DNAJB5 single gene GSEA results of DN. **(E)** The DNAJB5 single gene GSEA results of AS. **P<0.01, ***P<0.001.

**Figure 5:**
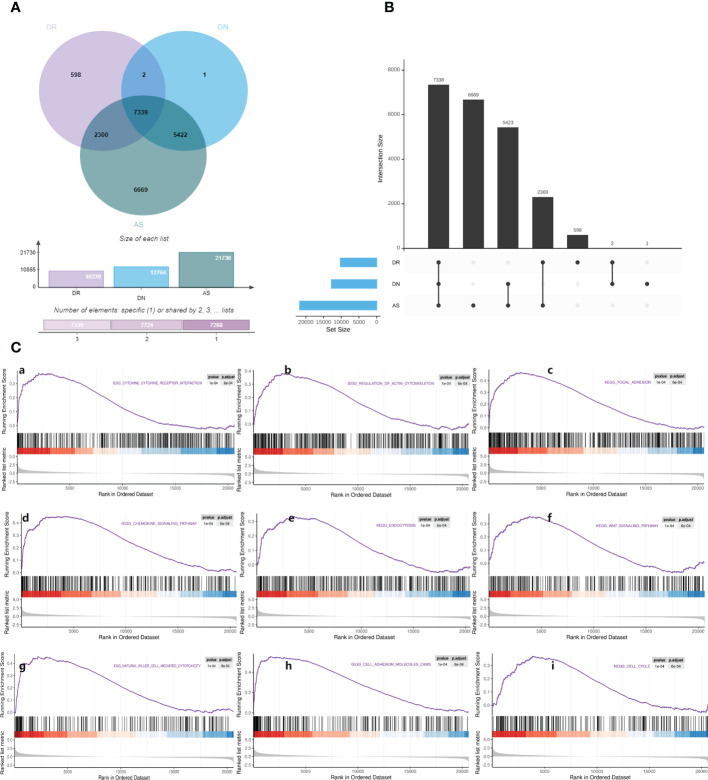
Gene set enrichment analysis. **(A)** Venn map shows the distribution of common differential genes in AS, DN, DR. **(B)** Upset map shows the cross linking of genes with significant differences among AS, DN, DR. **(C)** Gene set enrichment analysis: **(a)**. Enrichment plot of ‘CYTOKINE_CYTOKINE_RECEPTOR_INTERACTION’ with enrichment score 0.48, FDR q-value 0.0006. **(b)**. Enrichment plot of ‘REGULATION_OF_ACTIN_CYTOSKELETON’ with enrichment score 0.49, FDR q-value 0.0006. **(c)**. Enrichment plot of ‘FOCAL_ADHESION’ with enrichment score 0.59, FDR q-value 0.0006. **(d)**. Enrichment plot of ‘CHEMOKINE_SIGNALING_PATHWAY’ with enrichment score 0.56, FDR q-value 0.0006. **(e)**. Enrichment plot of ‘ENDOCYTOSIS’ with enrichment score 0.47, FDR q-value 0.0006. **(f)**. Enrichment plot of ‘WNT_SIGNALING_PATHWAY’ with enrichment score 0.53, FDR q-value 0.0006. **(g)**. Enrichment plot of ‘NATURAL_KILLER_CELL_MEDIATED_CYTOTOXICITY’ with enrichment score 0.56, FDR q-value 0.0006. **(h)**. Enrichment plot of ‘CELL_ADHESION_MOLECULES_CAMS’ with enrichment score 0.63, FDR q-value 0.0006. **(i)**. Enrichment plot of ‘CELL_CYCLE’ with enrichment score 0.56, FDR q-value 0.0006.

First of all, we searched for overlapping differential genes of three diseases (**sTable 1**), and found DNAJB5 (**Figure 4A**), a common significantly down regulated gene of three diseases (**Figure 4B**). Single gene GSEA analysis is used to explore biological functions related to DNAJB5 for AS, DN and DR(**Figures 4C–E**). In order to further explore the common differential genes and enriched biological functions of the three diseases (AS, DN, DR) from molecular clustering perspective, we first analyzed the differential genes of the three diseases by homonym aggregation. In order to ensure the integrity of the pathway and the integrity of the interaction relationship, we selected the common differential genes of the three diseases through Venn-upset plot and limited P-value<0.1. The results were shown in **Figures 5A, B**. In addition, all common DEGs expression levels in AS, DN and DR groups were applied to conduct GSEA functional analysis. As shown in **Figure 5C**, the GSEA analysis of common DEGs identified Cell cycle related pathway like regulation of actin cytoskeleton, chemokine signaling pathway, cell cycle, inflammatory related pathways like focal adhesion, endocytosis, natural killer cell mediated cytotoxicity, cell adhesion molecules cams, molecular medium action related pathway like cytokine-cytokine receptor interaction, WNT signaling pathway, ubiquitin mediated proteolysis.

### 3.5 Correlation analysis of multiple diseases

**Figure 6:**
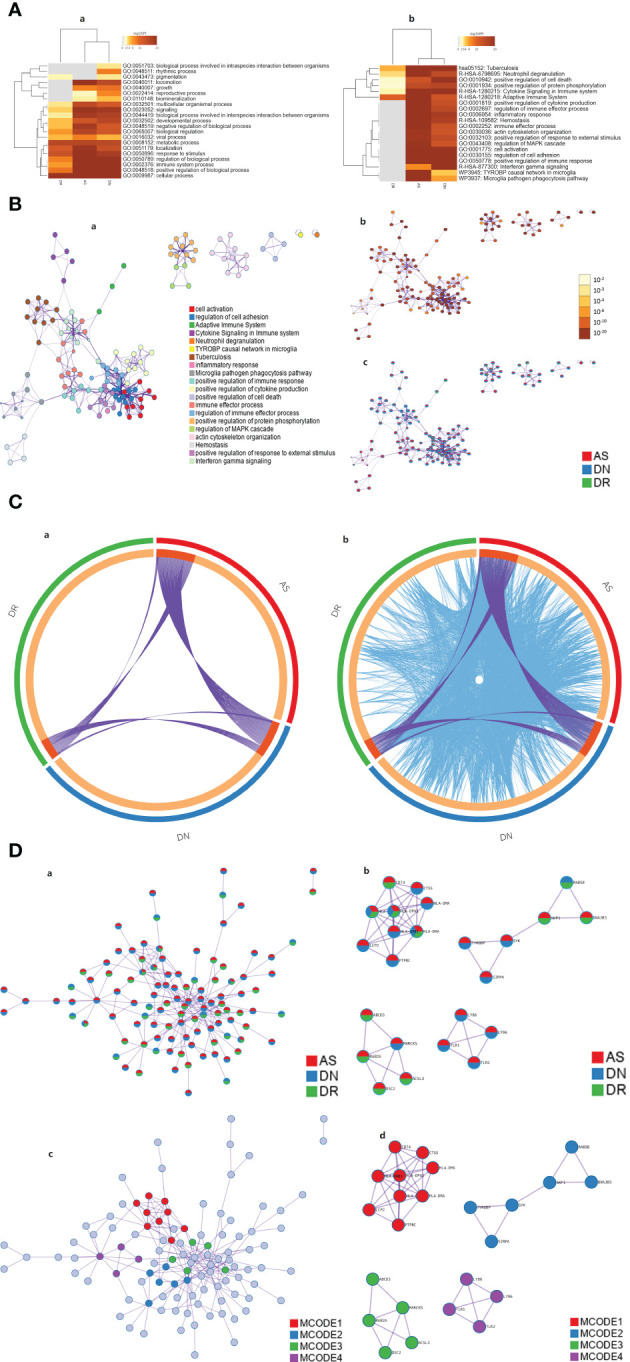
**(A)** Heatmap of enriched terms across input gene lists, colored by p-values. In GO pathway only [**(A)a**] and all pathway analysis [**(A)b**]. **(B)** Network of enriched terms: **(a)**. Colored by cluster ID, where nodes that share the same cluster ID are typically close to each other; **(b)**. Colored by p-value, where terms containing more genes tend to have a more significant p-value. **(c)**. Network of enriched terms represented as pie charts, where pies are color-coded based on the identities of the gene lists. **(C)** Overlap between gene lists: only at the gene level, where purple curves link identical genes **[(C)a]**; including the shared term level, where blue curves link genes that belong to the same enriched ontology term[**(C)b**]. **(D)** Protein-protein interaction network and MCODE components identified in the gene lists. **(a)**. All lists merged Colored by Counts (Full Connection). **(b)**. All lists merged Colored by Counts (Keep MCODE Nodes Only). **(c)**. All lists merged Colored by Cluster (Full Connection). **(d)**. All lists merged Colored by Cluster (Keep MCODE Nodes Only).

To comprehend the potential biological effects of the molecular differences between AS, DN and DR, DEGs of these three groups with log2 FCs ≥0.5 or ≤0.5 and P values <0.05 were identified between AS, DN, DR in the training cohort. The pathway and process enrichment analysis in the Metascape database showed that these DEGs were mainly enriched in the following pathways: multicellular organismal process, signaling, biological process involved in interspecies interaction between organisms, developmental process, negative regulation of biological process, biological regulation, viral process, metabolic process, localization, response to stimulus, regulation of biological process, immune system process, positive regulation of biological process, cellular process (GO terms); neutrophil degranulation, cytokine Signaling in Immune system, adaptive Immune System (Reactome Gene Sets); Tuberculosis (KEGG Pathway) (**Figure 6A**), then we constructed pathway network to get further understanding on pathway interaction (**Figure 6B**). After that, we defined the interaction and similarity relationship of disease sites through the Circos plot (**Figure 6C**), and the results showed that the correlations of significant sites of AS, DN, DR were remarkable. Finally, through the next step of analysis, PPI protein network interaction is obtained to further visualize gene information and build the PPI network for multiple diseases(**Figure 6D**). We use the MCODE plugin in Cytoscape to count the features of each node in the network graph, and the MCODE with the largest score value 1 was selected; genes in MCODE 1 were CD74, CTSS, LCP2, HLA-DRB1, HLA-DQB1,HLA-DPA1, HLA-DRA, HLA-DMA and PTPRC, which were mainly enriched in antigen processing and presentation of exogenous peptide antigen *via* MHC class II, as given in **Table 1**.

**Table 1:** Details of MCODE1.

Color	MCODE	GO	Description	Log10(P)
Red	MCODE_1	GO:0019886	antigen processing and presentation of exogenous peptide antigen *via* MHC class II	-19.8
Red	MCODE_1	GO:0002495	antigen processing and presentation of peptide antigen *via* MHC class II	-19.6
Red	MCODE_1	GO:0002504	antigen processing and presentation of peptide or polysaccharide antigen *via* MHC class II	-19.5

### 3.6 Identification of characteristic genes of diabetes

**Figure 7:**
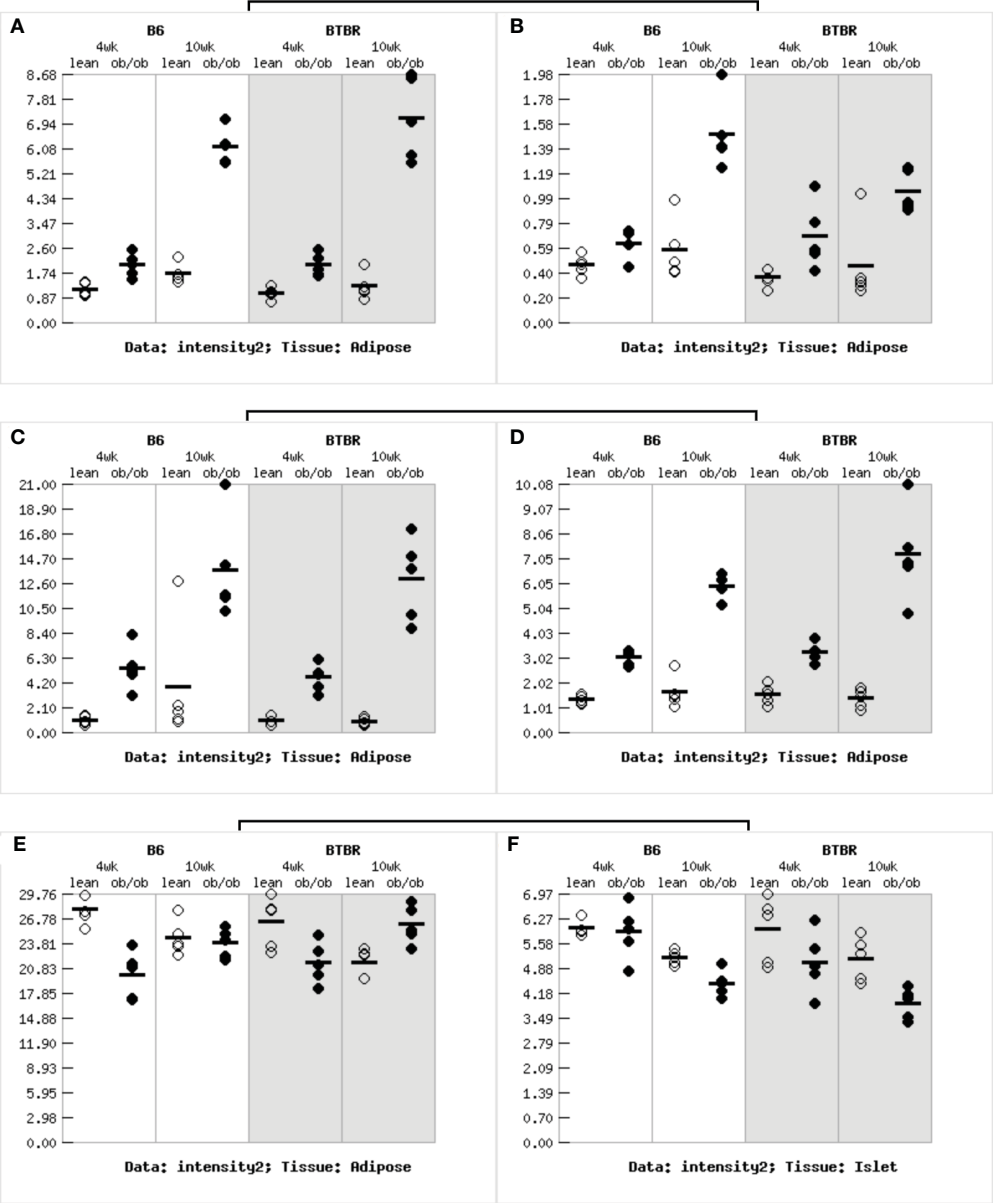
Gene expression in obese diabetes mice: **(A)**The expression of TYROBP gene in adipose tissue of obese diabetic mice. **(B)**The expression of LCP2 gene in adipose tissue of obese diabetic mice. **(C)**The expression of CCL2 gene in adipose tissue of obese diabetic mice. **(D)**The expression of CD44 gene in adipose tissue of obese diabetic mice. **(E)**The expression of RPL3 gene in adipose tissue of obese diabetic mice. **(F)**The expression of CDK4 gene in adipose tissue of obese diabetic mice.

First, we selected the central core sites in the PPI network of AS, DN, DR and found six characteristic genes of diabetes (TYROBP, LCP2, CCL2, CD44, RPL3, CDK4). To find the correlation between characteristic genes and diabetes, the Attie Lab Diabetes database was performed. B6 and BTBR mice were tested at 4 and 10 weeks of age, respectively. We examined the characteristic genes using the Attie lab diabetes database to determine the correlation between the characteristic genes from AS, DN, DR and diabetes. It was apparent that the expression of TYROBP, LCP2, CCL2, CD44 were significantly upregulated in the 4 and 10-week B6 and BTBR obese diabetic mice, while the expression of CDK4 were remarkably downregulated in the 4 and 10-weeks B6 and BTBR obese diabetic mice. The expression of RPL3, however, were intensely downregulated in the 4 and 10-weeks B6 obese diabetic mice and 4-weeks BTBR obese diabetic mice but upregulated in the 10-weeks BTBR obese diabetic mice (**Figure 7**).

### 3.7 Exploration of drug sensitivity for characteristic DEGs

**Figure 8:**
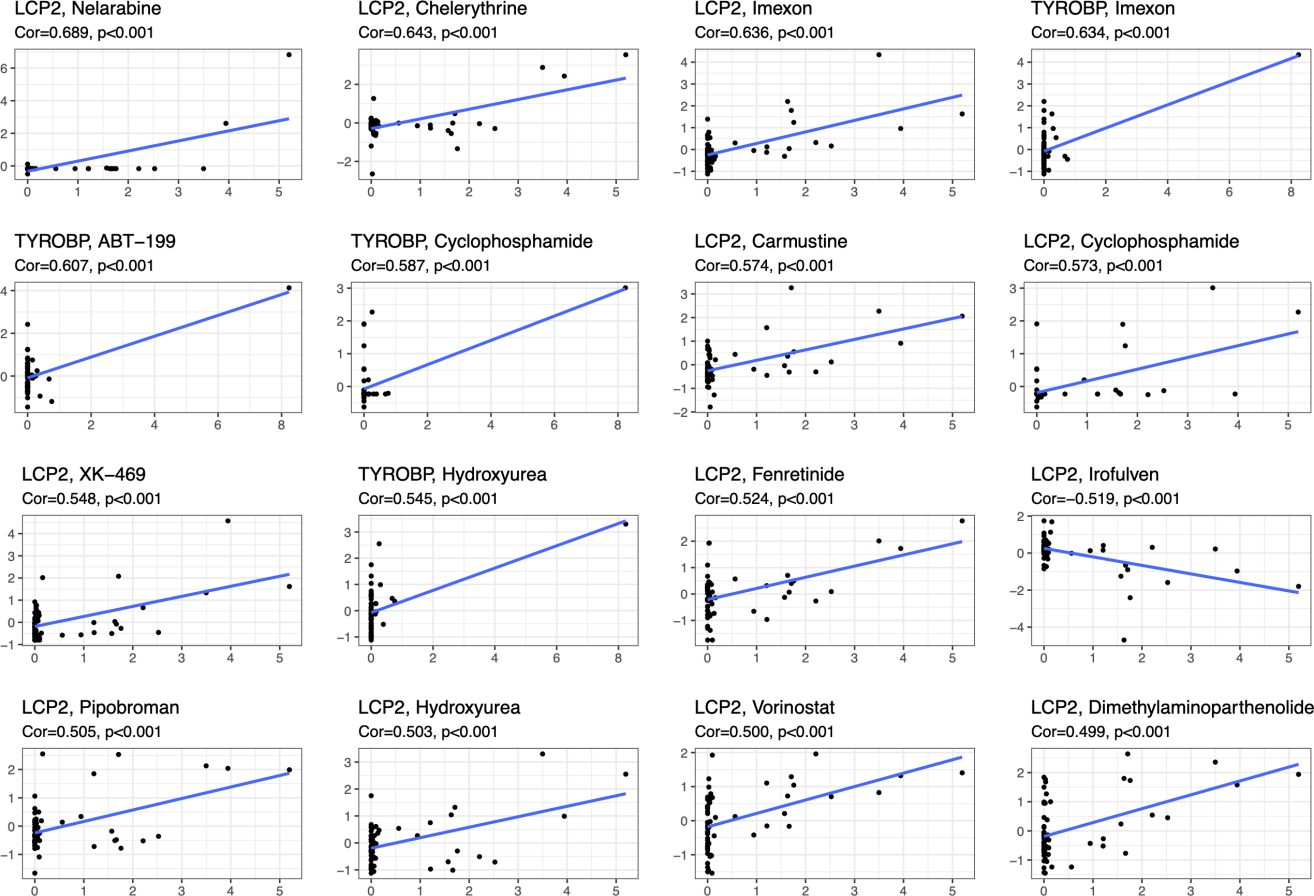
Correlation of drug sensitivity for characteristic DEGs.

We analyzed the drug sensitivity of characteristic DEGs, including TYROBP, LCP2, CCL2, CD44, RPL3, CDK4 (**Figure 8**; **sTable 1**). Vorinostat has significant positive correlation with LCP2, RPL3 and CDK4, but has negative correlation with CCL2. Drugs closely related to diabetes, such as Fenretinide, Dimethylamine parthenolide, Chelerythrine, etc., have obvious positive correlation with LCP2, TYROBP, RPL3, CDK4, and CD44. In addition, Cyclophosphamide and Hydroxyurea also show strong correlation with characteristic DEGs.

## 4 Discussion

Diabetes is often accompanied by vascular complications, including microvascular complications and macrovascular complications. Microvascular complications, such as DR and DN, are often considered to be identified before macrovascular complications, referring to AS and coronary heart disease. Furthermore, macrovascular complications are the primary cause of death in diabetes. Hence, we should attach great attention to the vascular complications of diabetes. Accumulating evidence demonstrated that diabetes related vascular complications can be attributed to several mainstream factors: hyperglycemia, hyperlipidemia, advanced glycation end products, growth factors and inflammatory cytokines/chemokines (14, 26).

In our study, the main joint functions of the two vascular complications of diabetes (three diseases: AS, DR, DN) can be summarized as: immunity, inflammation and oxidative stress. The results of our multi-disease correlation analysis show that the enrichment is highlighted in the function of MHC-II molecules. There is a widely recognized way of tissue damage in patients with diabetes, referring to abnormal lipid metabolism, which is also the reason for obesity in patients with diabetes. Cytokine production triggered by innate immune processes in adipose tissue macrophages (ATM) and adipocytes will drive adipose tissue inflammation in obesity and regulate the accumulation and polarization of ATM (27, 28). Studies signified that insulin resistance and AS can be prohibited by reducing the inflammation caused by adipocytes and ATMs (28–30). Adipocyte resident T cells (ART) are supposed to regulate fat inflammation and insulin resistance, and interact with adipocytes and ATMs (31). High glucose environment and abnormal fat metabolism will increase the number of CD8^+^T and CD4^+^Th1, leading to the secretion of IFN-γ by ART, which can promote adipocyte lipolysis and M1 polarization (32–34). Activating naive CD4^+^T to secrete cytokines and proliferate requires recognition of the specific peptide antigen (MHC-II complex) on antigen-presenting cells (APCs) and binding to costimulatory signals. Research indicated that the expression of class II trans activator (CIITA), the main transcriptional regulator of MHC-II pathway, and multiple MHC-II family genes (including costimulatory factors) increased significantly in adipocytes of obese subjects. A similar phenomenon was also observed in adipocytes of mice fed with high sugar and fat diet for 2 weeks. In addition, adipocytes were found to potentially activate T cells in contact dependent manner, while MHC-II deficiency attenuated inflammation and insulin resistance caused by a high sugar and fat diet (35). The above evidence elaborates that MHC-II molecule plays an important role in the inflammatory response involving adipocytes and immune cells. Combined with our analysis results, we can reasonably speculate that the immune inflammation caused by the accumulation of adipocytes and ATMs caused by lipid metabolism disorders will not only cause insulin resistance, but also damage the microvascular and macrovascular system in the high glucose and high fat environment, resulting in diabetes complications including AS, DR, DN.

Despite the immune inflammatory reaction caused by fat accumulation, chronic inflammation has been recognized as a major feature of diabetes complications. Simultaneously, there is evidence suggests us that inflammation is the first factor to be started-up before microvascular complications. In high glucose environment, endothelial cells are activated to express ICAM-1, VCAM-1 and E-selectin. These adhesion molecules promote leukocyte recruitment and extravasation, resulting in inflammation (36). Surveys have demonstrated that blocking or knocking down ICAM-1 can block the progression of DN (37). Concurrently, there is also evidence that down-regulation of ICAM-1 expression by neutralizing antibodies can improve atherosclerosis in rodent models (38). The above evidence supports our viewpoint that the massive secretion of adhesion factors caused by endothelial cell activation, which promotes the occurrence of inflammation, is one of the common causes of microvascular and macrovascular complications in diabetes. Under the chemotaxis of chemokines (CCL2, CX3CL1, CCL5, etc.), phagocytes such as monocytes and macrophages gather at the site of diabetes complications (39). After reaching and activating in the damaged tissue, monocytes and macrophages secrete a variety of factors (including Interleukin-1 β (IL-1 β) And CCL5) to promote the chemotaxis of other leukocytes such as T cells. There is evidence showing that excessive CCL-2 signal transduction and subsequent actin cytoskeleton reorganization will affect the expression of nephrin in glomerular podocytes, thus changing the structure and function of podocytes, leading to proteinuria (40, 41). In our annotation of the common functions of the three diseases (AS, DR, DN), the reorganization of the cytoskeleton of agonists is also very significant, which suggests that not only in DN, but also the excessive expression of chemokines and the transmission of chemotactic signals may lead to the occurrence of microvascular and macrovascular complications.

Many studies have identified five ways of vascular injury under high glucose environment, including specific inhibitors of aldose reductase activity, AGE formation, RAGE ligand binding, PKC activation, and hexosamine pathway. However, blocking any pathway alone against tissue damage in diabetes is far from satisfactory. Accordingly, some studies have proposed the hypothesis that these five mechanisms are caused by a large amount of ROS produced by upstream mitochondria. Superoxide is the initial oxygen free radical formed by mitochondria and then transformed into other more active substances, which can destroy cells in a variety of ways (42). In high glucose environment, it will reduce the activity of glyceraldehyde 3-phosphate dehydrogenase (GAPDH), a key glycolytic enzyme in cell types that cause intracellular hyperglycemia. When the activity of GAPDH is inhibited, the level of all glycolytic intermediates upstream of GAPDH will increase, thus activating five tissue damage pathways led by age and PKC (43, 44).

Chelerythrine, a benzophenanthridine alkaloid, is a potent, selective, and cell-permeable PKC inhibitor. Research shows that Chelerythrine can activate endogenous CSE/H2S through PKC/NF- κB, thereby reducing oxidative stress and protecting myocardial injury caused by renal ischemia-reperfusion injury in diabetes (45). Chelerythrine, the natural product, has been identified as a unique selective PPAR regulator (SPPARM) with strong PPAR γ binding activity. The mechanism of Chelerythrine in retaining the benefits of improving insulin sensitivity while reducing adverse reactions of thiazolidinedione shows that chelerythrine is a very promising drug, which can selectively target PPAR γ to further develop the clinical treatment of insulin resistance (46). Compared with the traditional ACEI drug (ramipril), dimethylaminoparthenolide not only achieves the efficacy of ramipril, but also significantly reduces glomerulosclerosis and tubulointerstitial fibrosis (47). Dimethylamine parthenolide is considered as a compound with potential added value for ACEI treatment of DN. The results obtained through the drug sensitivity of characteristic DEGs allow us to broaden the horizon of treatment of microvascular complications of diabetes from the level of comorbidity.

We all know that DR can visually observe the changes of fundus blood vessels through ophthalmoscopy, and the non-invasive screening of DN mostly comes from routine urine examination. If we want to directly observe the pathological changes of DN, we need to have traumatic renal puncture. AS, a macrovascular disease, often has no obvious clinical symptoms in the early stage, but with the progress of the disease, unhealthy macrovascular may threaten the lives of patients. Therefore, we try to clarify the common mechanism between microvascular and macrovascular complications of diabetes, exploring its intrinsic relationship, so as to find potential prevention and treatment targets, and strive to sensitively detect the existence of life-threatening complications in routine examinations, which is of great significance for prolonging the survival time of patients with diabetes and improving their quality of life. The exploration of disease pathway interactions *in vivo* and *in vitro* is essential to describe the specific role of the identified pathogenesis, which may help to identify key co-pathogenesis hubs and reveal the potential mechanism of the development of these two types of vascular complications (three diseases).

## 5 Conclusion

In conclusion, in the microvascular and macrovascular complications of diabetes, immune-mediated inflammatory response, chronic inflammation caused by endothelial cell activation and oxidative stress are the three component linking AS, DN and DR together. Clarify he intrinsic relationship and common tissue injury mechanism of microcirculation and circulatory system complications in diabetes and looking for the mechanism hub of the two types of vascular complications have far-reaching clinical and social value for reducing the incidence of fatal events and early controlling the progress of fatal and disabling complications of circulatory system in diabetes.

## Data availability statement

The original contributions presented in the study are included in the article/**Supplementary Material**. Further inquiries can be directed to the corresponding authors.

## Author contributions

**F**M and YG conceived the study, critically reviewed the intellectual content of the manuscript and made substantive revisions to the important contents of the manuscript. ZH, CaY and MF were the major contributor to the research and the writing of the manuscript. DQ, CL and GX provided technical support and revised the manuscript. ChY, YX and XZ provided valuable suggestions. All authors contributed to the article and approved the submitted version.
