# Supplementary material for: Exploring the shared molecular mechanism of microvascular and macrovascular complications in diabetes: Seeking the hub of circulatory system injury
Source: Front Endocrinol (Lausanne). 2023 Jan 23;14:1032015. doi: 10.3389/fendo.2023.1032015 (PMC9899888; doi:10.3389/fendo.2023.1032015)
Supplement: Supplementary file 3 [file DataSheet_3.docx]

**
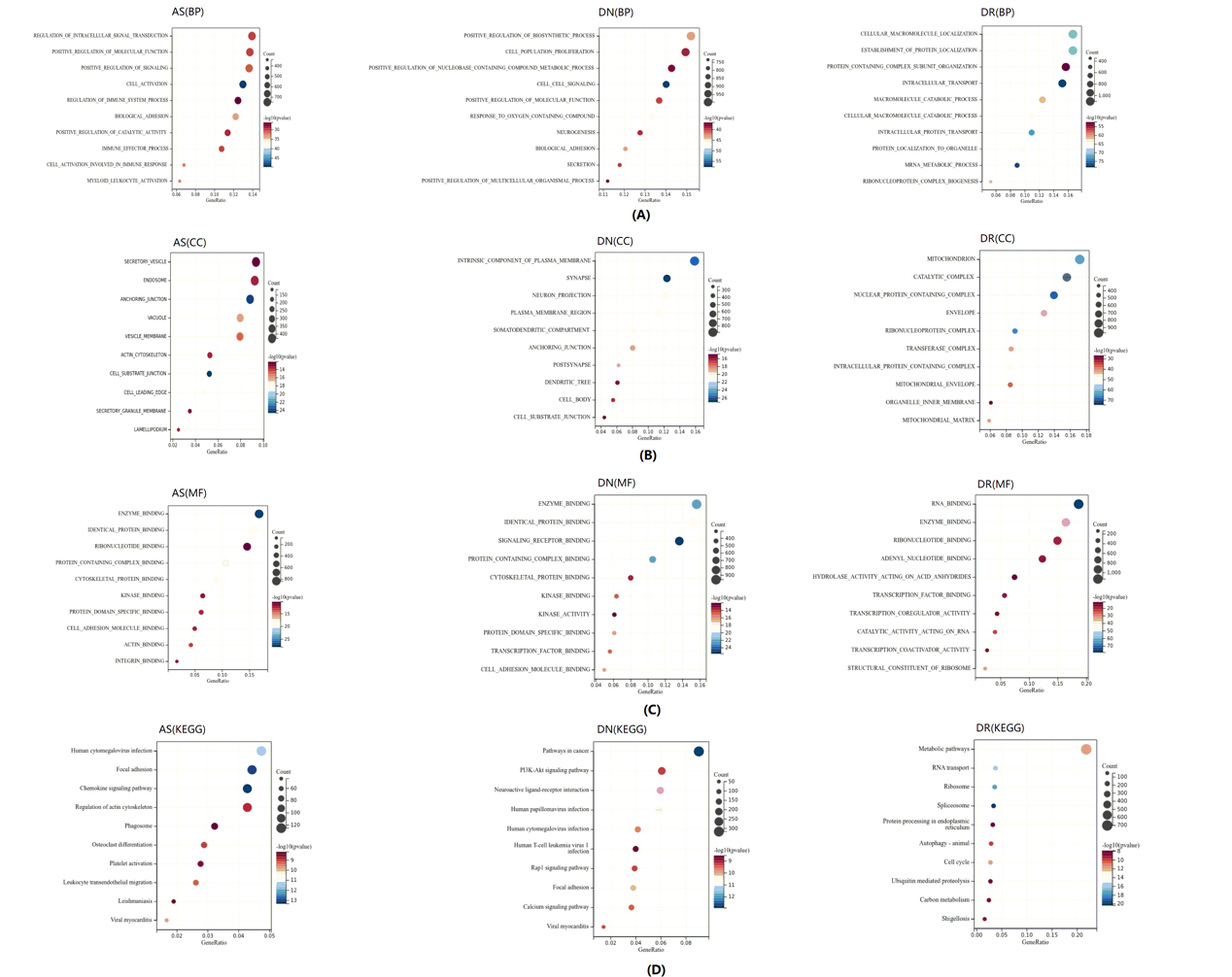
**

**S_Figure. 1：GO and KEGG pathway enrichment analysis of DEGs. (A) GO enrichment analysis on the Biological Process (BP) terms of DEGs in AS, DN and DR. (B) GO enrichment analysis on the Cellular Component (CC) terms of the DEGs in AS, DN and DR. (C) GO enrichment analysis on the Molecular Function (MF) terms of the DEGs in AS, DN and DR. (D) KEGG terms in the enrichment analysis of the DEGs in AS, DN and DR.**
